# Room-Temperature Adsorption on Magnetite Induces Spontaneous Fragmentation of Early Transition-Metal Size-Selected Nanoclusters

**DOI:** 10.1021/jacs.6c15419

**Published:** 2026-09-17

**Authors:** Gabriele Coltrioli, Deborah Perco, Luca Sementa, Marco Bianchi, Andrea Berti, Mikhail Bandurist, Paolo Lacovig, Silvano Lizzit, Aras Kartouzian, Ueli Heiz, Alessandro Fortunelli, Alessandro Baraldi

**Affiliations:** † Department of Physics, 9315 University of Trieste, Via Valerio 2, 34127 Trieste, Italy; ‡ Istituto per i Processi Chimico-Fisici, 9327 Consiglio Nazionale delle Ricerche, Via Giovanni Moruzzi 1, Pisa 56124, Italy; § Elettra - Sincrotrone Trieste, S.S. 14 Km 163.5, AREA Science Park, 34149 Basovizza-Trieste, Italy; ∥ Chemistry Department & Catalysis Research Center, Technical University of Munich, Lichtenbergstrasse 4, Garching D-85748, Germany; ⊥ Istituto di Chimica dei Composti Organometallici, Consiglio Nazionale delle Ricerche (CNR-ICCOM), Via Giovanni Moruzzi 1, 56124 Pisa, Italy

## Abstract

The stability of supported metal nanoclusters, namely, their ability to preserve structure, composition, and catalytic activity over time, is a fundamental prerequisite for catalyst durability, resistance to deactivation, and long-term performance under operating conditions. Despite their often-exceptional intrinsic activity, nanoclusters are particularly susceptible to structural evolution driven by strong metal–support interactions. Here, we demonstrate that size-selected early transition-metal nanoclusters (W, Mo, and Ta) soft-landed on reconstructed Fe_3_O_4_(001) undergo spontaneous breakdown already at room temperature, in the absence of thermal activation or reactive gas environments. Combining high-energy resolution photoelectron spectroscopy and scanning tunneling microscopy with density functional theory, we show that strong metal–oxygen bonding, together with reconstruction-induced surface defects of the oxide support, provides the driving force required to overcome metal–metal cohesion within the clusters, leading to their dissociation. Upon further oxidation, an enhanced degree of atomization is produced, particularly for larger clusters, yielding highly dispersed metal species. The propensity for fragmentation, i.e., the partial or total breaking of metal–metal bonds in the cluster, follows the trend Ta > W > Mo, which correlates closely with metal oxophilicity, adsorption energies, and intrinsic metal–metal bond strengths. These findings establish adsorption-induced fragmentation of metal nanoclusters as an intrinsic manifestation of metal–support interactions for early transition metals on reducible oxides. More broadly, they reveal a spontaneous room temperature pathway to cluster destabilization and identify an innovative strategy for the controlled generation of high densities of single metal atoms from well-defined nanocluster precursors.

## Graphical abstract

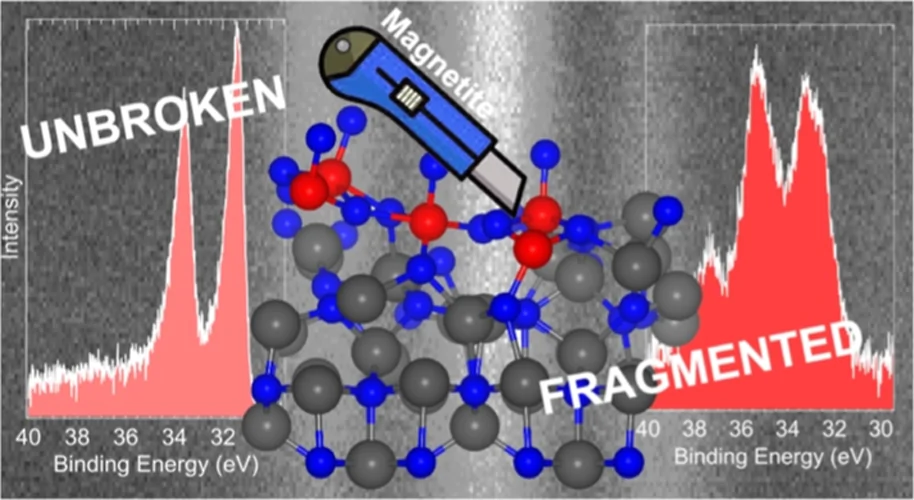

## Introduction

It is well known that under reaction conditions, supported metal clusters and nanoparticles can undergo agglomeration into larger particles via Ostwald or Smoluchowski ripening, at the expense of smaller aggregates, reducing the number of active sites and their efficiency.^1–6^ Considerable efforts have therefore been focused on studying the sintering process^7–14^ in supported atomic clusters, a crucial class of materials whose chemical properties are strongly dictated by their size and geometric structure^15–18^ with the final aim to achieve surface stabilization. This achievement, together with catalytic activity and selectivity, becomes central when assessing the viability of a catalyst for practical applications. A second important catalytic deactivation mechanism is cluster fragmentation.^19^ For instance, Pt clusters supported on CeO_2_ are converted into single atoms under propane-oxidation conditions, generating spectator species with markedly reduced chemical reactivity.^20^ Similarly, γ-Al_2_O_3_-supported Pd nanoparticles are known to rapidly decompose into inactive single atoms during methane oxidation.^21^ It is essential to emphasize that the detachment of single atoms from a cluster is a thermally activated process, requiring high temperatures, consistent with the intrinsically high cohesive energies of metal clusters. Indeed, depending on the support, the conversion of metal clusters into smaller aggregates under reaction conditions can take place even after sintering and at very high temperatures. As recently reported by Wei et al.,^22^ single atoms can be generated from the decomposition at 900 °C of noble metal nanoparticles on nitrogen-doped carbon substrates, exhibiting superior activity and selectivity in the semi-hydrogenation of acetylene.

The importance of cluster fragmentation is further underscored by the significant synergistic effects that can arise when single atoms and clusters coexist as these combinations can strongly influence the catalytic properties of a material. An illustrative example is provided by the case in which Pt–Ti single atoms coexist with Pt nanoparticles on defective TiO_2_, leading to enhanced O_2_ activation.^23^ In this system, the lattice distortion induced by the alloyed Pt–Ti single atoms further promotes the activation of neighboring lattice oxygen, enabling low-temperature CO oxidation. Similar cooperative behaviors have been observed in other systems, as well. Ma et al. showed that Fe atoms and nanoclusters supported on *N*-doped porous carbon act synergistically in the aerobic oxidation of primary amines,^24^ while Gao et al. demonstrated that Pd atoms and clusters on CdS concurrently serve as oxidation and reduction sites during methanol dehydrogenation.^25^

To date, however, fragmentation has been observed only under specific conditions, typically driven by high temperatures or adsorbates, and no evidence has yet shown whether similar processes can occur under milder reaction environments. Recent theoretical work has highlighted that, for late transition metals and coinage metals, the interaction of the supported metal with the metal of the support can dominate over the influence of the bond with oxygen and can drive the encapsulation of clusters and nanoparticles.^26^ It is also worth noting that even single metal atoms can play a significant role in promoting the reduction of oxide surfaces, including those considered to be irreducible oxides such as zirconia. In this context, it has been even proposed that the reducibility of such oxides can be rationally tuned by appropriately selecting the dispersed transition-metal atoms.^27^

Nevertheless, our understanding of the synergistic roles played by all the various contributing factors, such as substrate nature, metal–adsorbate interactions, metal–metal interactions, and temperature in determining whether and how clusters and particles fragment remains limited.

In this work, we demonstrate that metal–support interactions induce the spontaneous fragmentation of atomically precise early transition metal nanoclusters (W, Ta, and Mo) supported on Fe_3_O_4_(001) without any external driving force such as elevated temperature or adsorbates. The unique structure of the ( $\sqrt{2}x\sqrt{2}$ )­R45° reconstructed surface of magnetite, which recently emerged as an excellent support for single atoms, featuring the presence of interstitial Fe sites in the second layer, favors the bonding of highly stable atoms.^28–31^ By combining high-resolution X-ray photoelectron spectroscopy (HR–XPS) and density functional theory (DFT) calculations, we show that the presence of spectral components in W 4f, Mo 3d, and Ta 4f core level spectra corresponding to highly oxidized species, even for the larger clusters, as well as the absence of any metal-induced spectral features, can be linked to cluster fragmentation. Most importantly, this process of cluster disgregation upon deposition takes place already at 300 K. This fragmentation effect is particularly pronounced for Ta clusters, which show the formation of a high density of single atoms already upon deposition. A decisive role for the room temperature breakup of clusters is played by the defects present on the reconstructed magnetite surface. Indeed, an ideally bulk-terminated Fe_3_O_4_(001) surface exhibits a much lower tendency to induce nanocluster dissociation. Subsequent oxygen exposure of W, Ta, and Mo nanoclusters resulted in further changes in the relative intensities of the spectral components only for large sizes, which is attributed to a further atomization of clusters.

## Results and Discussion

Size-selected *W*_n_ clusters (*n* = 1, 3, 5, 7, 13, and 25) were generated and deposited in situ on freshly prepared, reconstructed Fe_3_O_4_(001). Over the past decade, this surface has been extensively studied, demonstrating that the surface reconstructs according to the subsurface cation vacancy (SCV) model and is widely used as a support for single metal atoms.

Figure 1a shows the characteristic $\left(\sqrt{2}\times \sqrt{2}\right)$ R45° low-energy electron diffraction (LEED) pattern of the clean Fe_3_O_4_(001) surface. The presence of a squared unit cell is evidenced by the reciprocal unit vectors, labeled as a⃗_1_* and a⃗_2_*. The appearance of the SCV reconstructed phase is fully validated by our LEED-IV analysis, which yields an excellent Pendry R-factor of 0.16 (see Figure S6 Supporting Information). Panel (b) illustrates the characteristic undulating, zigzag-like appearance of the surface Fe_oct_ rows, which originates from the SCV reconstruction. Within the SCV model, this reconstruction entails a rearrangement of the subsurface cation lattice wherein two octahedral Fe cations are replaced by a single interstitial Fe cation, thereby lowering the surface energy and breaking the symmetry of the bulk termination. Consequently, two non-equivalent sites, typically designated as “wide” and “narrow,” are formed between adjacent Fe_oct_ rows within each unit cell. These distinct local coordination environments induce alternating lateral relaxations of the surface Fe_oct_ atoms toward or away from neighboring rows, driven by the presence or absence of a cation vacancy in the second subsurface layer. Furthermore, panel (c) highlights domain boundaries separated by monoatomic steps, while panel (d) reveals a locally unreconstructed unit cell that appears as a pair of bright protrusions along adjacent Fe_oct_ rows. As discussed in ref 32, this defect corresponds to a region where an excess Fe atom and the interstitial Fe atom associated with the SCV reconstruction occupy bulk-like positions within the spinel lattice, locally restoring the bulk termination and suppressing the reconstructed geometry.

**Figure 1:**
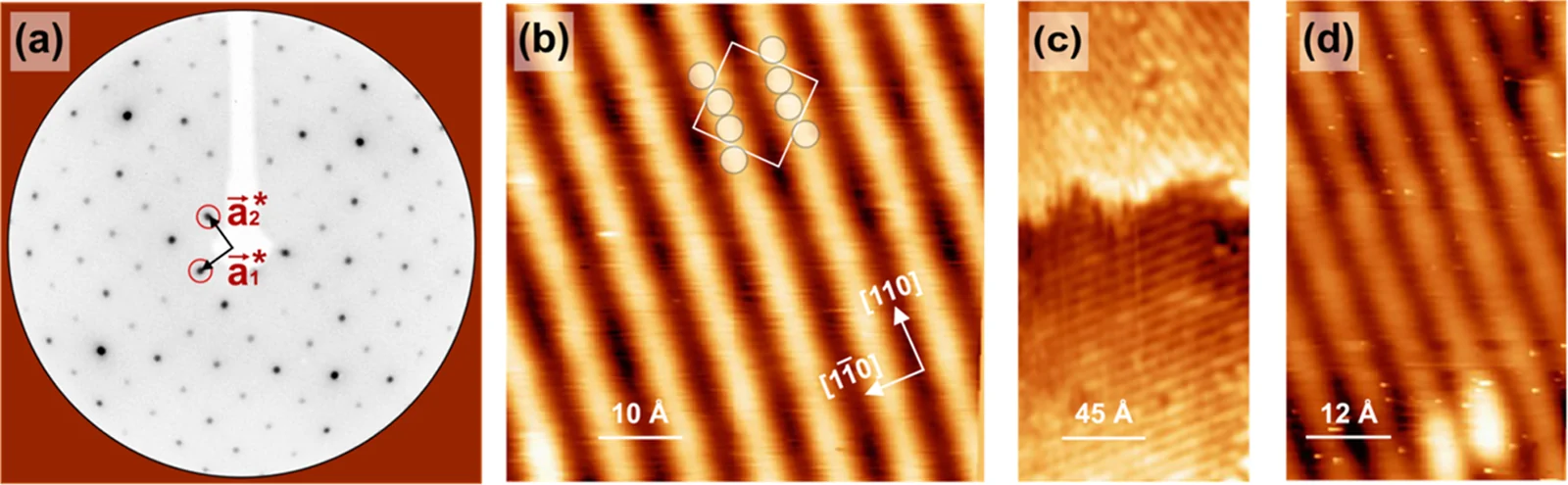
Structural characterization of the Fe_3_O_4_(001) SCV-reconstructed surface. (a) LEED pattern (*E* = 148 eV) of the clean Fe_3_O_4_(001) surface showing the characteristic $\left(\sqrt{2}\times \sqrt{2}\right)$ R45°reconstruction. The reciprocal unit vectors a⃗_1_* and a⃗_2_* define the reconstructed surface unit cell. (b) Scanning tunneling microscopy (STM) image (*V*_bias_ = 1.4 V, *I*_tunnel_ = 0.1 nA, 46 × 46 Å^2^) highlighting the characteristic zigzag modulation of the surface Fe_oct_ rows induced by the SCV reconstruction, with inequivalent wide and narrow sites within the unit cell. (c) STM image (*V*_bias_ = 1.4 V, *I*_tunnel_ = 0.1 nA, size 124 × 263 Å^2^) showing reconstructed domains separated by monoatomic steps. (d) STM image (*V*_bias_ = 1.4 V, *I*_tunnel_ = 0.1 nA, size 40 × 70 Å^2^) of a locally unreconstructed unit cell, identified by the characteristic pair of bright protrusions associated with a defect region where the bulk-like Fe_3_O_4_(001) termination is locally restored.

Parkinson and co-workers showed that Au adatoms on this substrate remain stable against sintering up to 673 K^31^, while Ag^28^ adatoms are stable as long as the coverage remains below 0.5 ML. In general, the tendency for atomic incorporation increases from right to left across the transition metal series and from heavier to lighter elements within the same group. For example, Co^33^ and Ni^34^ adatoms partially diffuse into subsurface sites, whereas Rh^29^ can exist either as single atoms or as nanoparticles depending on the coverage. Among supported metal clusters, however, only Pt has been systematically investigated on this surface.^35,36^ Fe_3_O_4_(001) was therefore selected to examine how this substrate influences the geometrical and electronic properties of early transition metal clusters. Cluster sizes *n* = 13 and *n* = 25 were chosen for a comparison with previous measurements of W_13_ and W_25_ supported on graphene/Ru(0001).^37^ In order to follow the evolution from very small to more bulk-like clusters, clusters containing 1, 3, 5, and 7 atoms were also studied. Aiming to reduce temperature-induced effects, particularly diffusion and sintering, all of the clusters were deposited at 300 K. Mass selection was verified by acquiring mass spectra, reported in Figure S1 of the Supporting Information To preserve the mass selection, we performed deposition in the soft-landing regime. The resulting cluster density was kept below 1 × 10^–2^ clusters per nm,^2^ further ensuring clear mass selection. Ta monomers were produced by means of the same mass selection process, yielding a coverage of 0.6% ML. In contrast, monomeric W and Mo species were produced using an evaporator leading to a final coverage of respectively 0.3% of ML and 2.9% of ML (1 ML = 1.42 × 10^14^ atoms/cm^2^). HR-XPS measurements were carried out at the SuperESCA beamline of Elettra (see the Experimental Methods section).

Figures 2a and b illustrate the morphological characteristics of W single atomic species following evaporation-based deposition. The two panels present STM images acquired under W evaporation conditions identical to those employed in our photoemission experiments. These data reveal the appearance of discrete, bright features with an apparent height of approximately 2 Å, with high-resolution imaging located precisely between the Fe_oct_ rows of the reconstructed magnetite surface. The lateral extent and registry of these features are consistent with W atoms incorporated in the surface, mirroring the behavior observed for other transition metals on the Fe_3_O_4_(001) surface.^38^ These findings confirm that our evaporation-based deposition of monomers successfully produces surface-incorporated atomic species, bridging the structural link between the observed oxidation states and the atomic-scale geometry of the SCV-reconstructed substrate.

**Figure 2:**
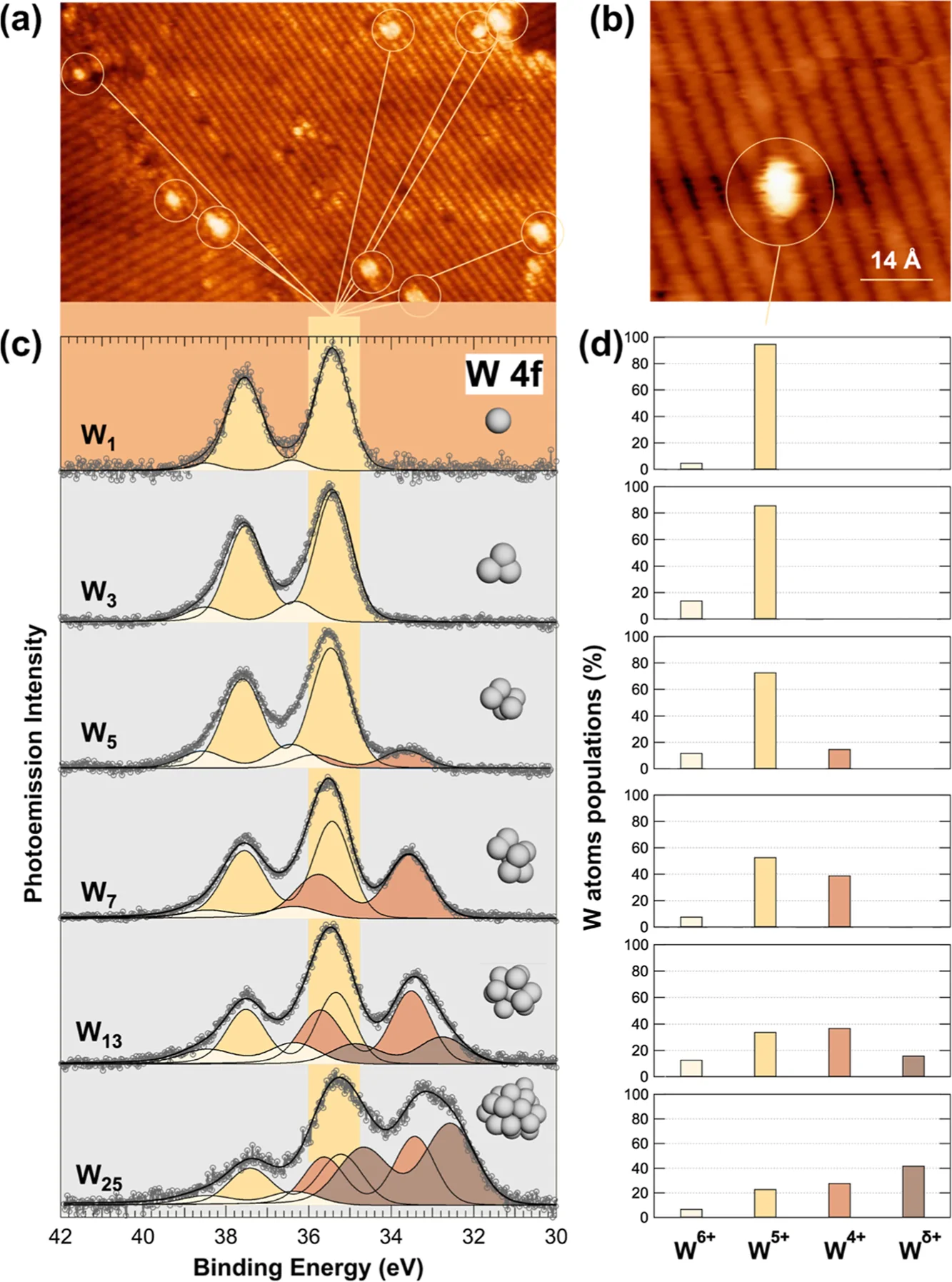
(a) STM image (*V*_bias_ = 1.4 V, *I*_tunnel_ = 0.1 nA, 240 × 145 Å^2^) illustrating the morphological characteristics of isolated W species following evaporation-based deposition, acquired under conditions identical to those used in the photoemission experiment in panel (c). (b) High-resolution STM image (*V*_bias_ = 1.4 V, *I*_tunnel_ = 0.1 nA, 60 × 60 Å^2^) zooming on one bright feature with an apparent height of approximately 2 Å, located precisely between the Fe_oct_ rows of the reconstructed Fe_3_O_4_(001) surface. The lateral registry of these features is consistent with W atoms on the surface. (c) Fitted W 4f core level spectra of W_n_ nanoclusters. The experimental curves are displayed as grey markers, and the fitting curves as black lines. The spin–orbit doublets corresponding to W atoms in different oxidation states are shown in various colors: beige (W^6+^), orange (W^5+^), red (W^4+^), and brown (W^δ+^). (d) Histograms of the percent areal contributions of W atomic populations to the total spectra area as a function of the W-oxidation state. The bars are displayed with the same colors used for the spin–orbit doublets in panel (a).

Figure 2c shows the W 4f core level spectra of pristine W clusters after background subtraction (see Experimental Methods for details), along with their corresponding fits. All the spectra were analyzed using Doniach–Šunjić lineshape convoluted with a Gaussian. For the smallest clusters, W_1_(monomer) and W_3_(trimers), the characteristic two spin–orbit doublets (Δ*E* = 2.15 eV) were consistently observed, with W 4f_7/2_ binding energies in the ranges of 35.2–35.4 eV and 36.3–36.4 eV. Although in the case of nanoclusters it is not straightforward to make a direct association between spectral components and oxidation states these values are close to those of WO_3‑x_ and WO_3_, suggesting that they can be attributed to W^5+^ (orange ones in Figure 2c) and W^6+^ (beige) species. As the cluster size increases, additional components appear at lower binding energies. W_5_ and W_7_ exhibit a third doublet with W 4f_7/2_ at 33.4–33.7 eV, consistent with the binding energy of WO_2_, while W_13_ and W_25_ require an additional component at 32.5–32.7 eV. These two new features were assigned to W^4+^ and W^δ+^ (0<δ < 4), represented in red and brown in Figure 2c.

Two observations are particularly worth noting. First, even W monomers exhibit two spectral components, although one is much weaker in intensity. This behavior contrasts with metals such as Pt, Ag, and Ir, whose monomers typically display only a single component in their core level spectrum on the same substrate.^28,30,39^ In contrast, Co_1_/Fe_3_O_4_(001) and Ni_1_/Fe_3_O_4_(001) show two components associated with different oxidation states, while Rh_1_/Fe_3_O_4_(001) exhibits a variable number of components depending on the coverage.^29,33,34^ These multiple features have been attributed to adatoms occupying different adsorption sites including subsurface positions.

By analogy, the two components observed for W_1_ likely correspond to atoms occupying distinct local geometries on Fe_3_O_4_(001). The second noteworthy point is the following: as shown in Figure 2d, even the spectra of the largest clusters, W_13_ and W_25_, are dominated by features associated with highly oxidized species, and no spectral signature of metallic W is detected. This behavior suggests either (i) a substantial charge transfer from the substrate to the clusters or (ii) a significant geometric rearrangement, such as fragmentation, driven by strong metal–substrate interactions. It is important to emphasize that this trend contrasts sharply with the spectra previously reported for W_13_ and W_25_ supported on graphene/Ru(0001),^37^ prepared under identical soft-landing conditions and base pressure, where the spectra were instead dominated by features attributed to metallic W (BE = 31.3–31.4 eV). We therefore conclude that the pronounced oxidation observed here originates already upon deposition from the interaction with the oxide substrate.

After deposition, the W clusters were exposed to molecular oxygen at increasing pressures, from 1 × 10^–9^ to 1 × 10^–6^ mbar, for a total exposure of 436 L. The evolution of the W 4f core level spectra was monitored continuously during the exposure, allowing a real-time tracking of the cluster’s oxidation process. Figure 3a shows the spectra acquired at the beginning and end of the oxygen exposure, together with a gray scale representation of the photoemission intensity as a function of binding energy and oxygen exposure, highlighting the evolution of the individual components during the process.

**Figure 3:**
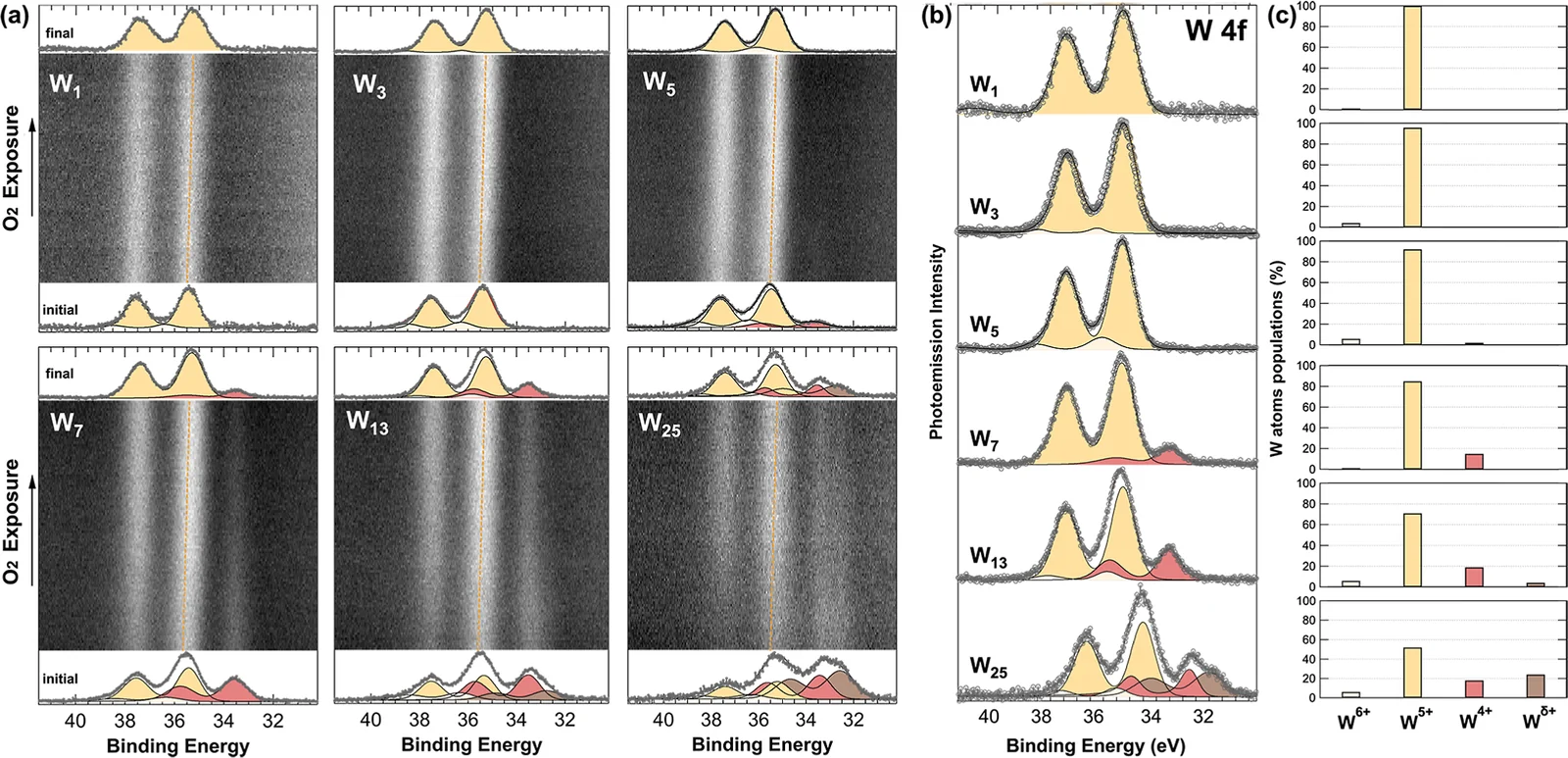
(a) Two-dimensional maps of the evolution of W 4f core level spectra for different clusters during oxygen exposure. Photoemission intensity (from low to high) is presented in gray scale (from black to white) respectively as a function of binding energy and oxygen exposure. The initial and final core level spectra are reported with their fitted spin–orbit doublets, below and above each map, respectively. (b) Fitted W 4f core level spectra of *W*_n_ nanoclusters after exposure to 436 L of O_2_. The spin–orbit doublets corresponding to W atoms in different oxidation states are shown in various colors: beige (W^6+^), orange (W^5+^), red (W^4+^), and brown (W^δ+^). (c) Histograms of the percent areal contributions of W atomic populations to the total spectra area as a function of the W-oxidation state. The bars are displayed with the same colors used for the spin–orbit doublets in panel (b).

A general trend is immediately evident for all cluster sizes: the W^5+^ component shifts to lower binding energy by approximately 0.2 eV (red guide to the eye lines in Figure 3a). This shift, which will be discussed in more detail later, is likely associated with structural rearrangements occurring during the oxygen exposure. Apart from this shift, no new components emerge during oxidation and only spectral modifications involve variations in the relative intensities of the existing components. While W_1_ and W_3_ show almost no spectral changes, from W_5_ onward, the component assigned to W^5+^ increases markedly in intensity at the expense of lower oxidation state species. It is also evident that smaller clusters reach saturation more rapidly: for example, W_5_ saturates after approximately 20 L exposure, whereas W_25_ requires roughly 50 L.

Figures 3b and c display the W 4f core-level spectra acquired after the oxidation and the histograms of the relative atomic populations, respectively.

Compared to Figure 2, oxygen exposure clearly leads to the predominance of the W^5+^ component for all cluster sizes. For the smaller ones, W_1_ and W_3_, the highest binding energy component, attributed to W^6+^, almost disappeared. For larger clusters, the lowest binding energy components are progressively converted to the higher oxidation-state ones. The shift toward lower BE, indicated as slightly tilted dotted lines in panels of Figure 3a, is more pronounced for W^5+^ and W^6+^ components, whereas the others shift only slightly.

Aiming at a deeper understanding of the experimental observations and to identify the origin of the absence of any metallic component in the core level spectra, DFT calculations were performed. The Fe_3_O_4_(001) surface was modeled as a slab using the ( $\sqrt{2}x\sqrt{2}$ )­R45° unit cell. W_1_, W_3_, and W_5_ were selected as model clusters, and their global minimum structure was predicted via Density-Functional Global Optimization (GO) simulations.^40^ The top and side views of the GO-optimized geometries are reported in Figure 4a. W_1_ adopts a peculiar structure in which the W atom substitutes one of the surface Fe cations with octahedral coordination (Fe_oct_), achieving fivefold O coordination, like the one in bulk W oxides. The substitution also displaces a neighboring Fe_oct_.

**Figure 4:**
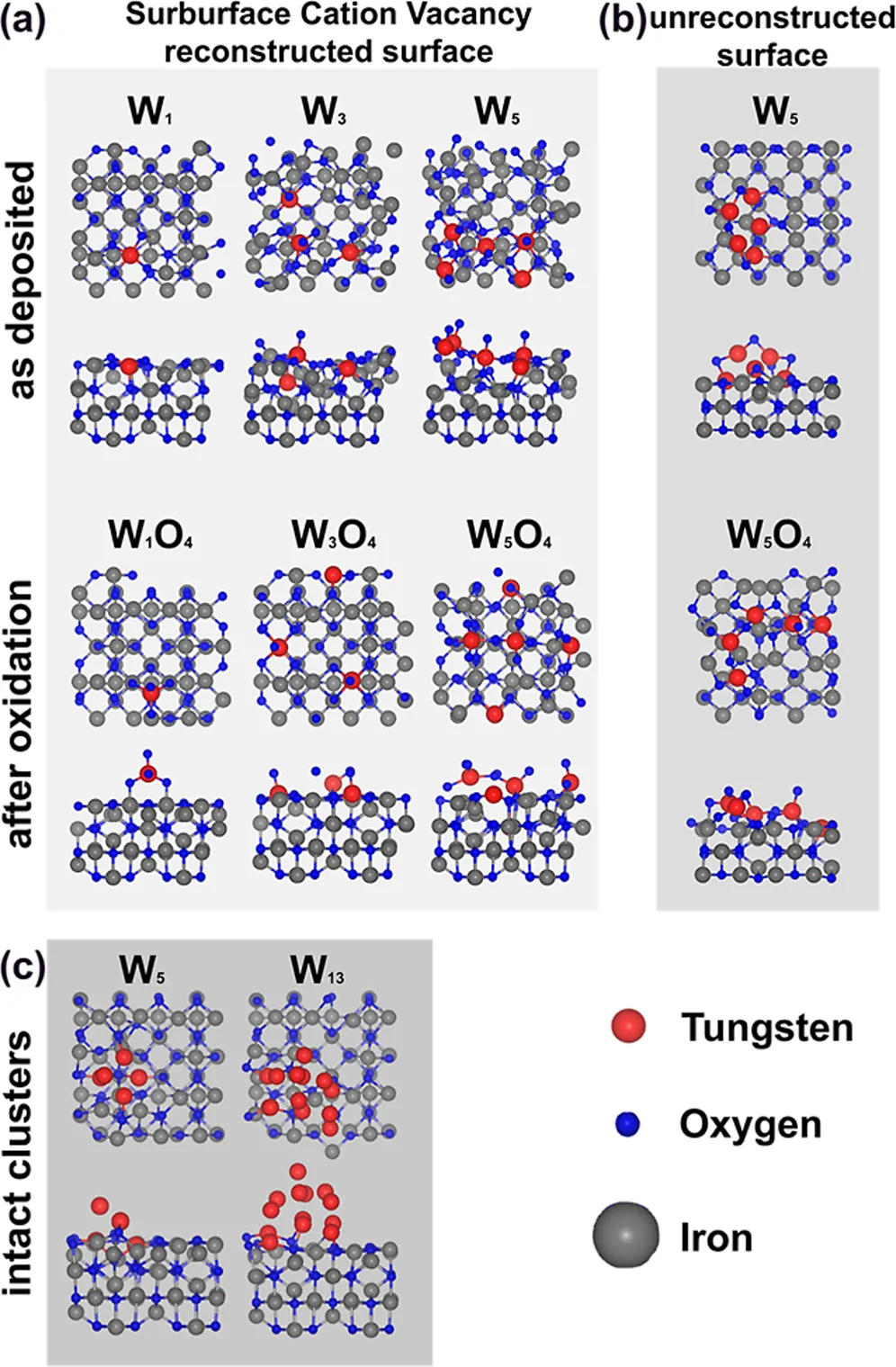
(a) Top and side views of the DFT@GO-optimized configurations of W_1_, W_3_, and W_5_ clusters on SCV-reconstructed model of the Fe_3_O_4_(001) surface, both for pristine clusters and after addition of 4 oxygen atoms. (b) Top and side views of the DFT@GO-optimized configurations of the W_5_ cluster on non-reconstructed and reconstructed Fe_3_O_4_(001) surfaces, both for pristine clusters and after addition of 4 O atoms. (c) Minimum-energy configurations of intact W_5_ and W_13_ clusters absorbed on the SCV-reconstructed Fe_3_O_4_ (001) surface, obtained by placing the gas-phase global-minimum geometries on the surface and subsequently relaxing the structures.

A similar behavior is also observed for W_3_: two atoms of the trimer replace two central Fe_oct_ cations in the first layer, while the third one penetrates the subsurface and replaces an Fe_oct_ in the third layer, as the second-layer Fe cations with tetrahedral coordination (Fe_tet_) are entirely displaced. These results highlight the strong tendency of W to maximize oxygen coordination, preferentially replacing Fe_oct_ rather than Fe_tet_. W_5_ shows analogous behavior, with W atoms replacing first-layer Fe_oct_, resulting in a significantly disordered surface. A key finding concerns the metal–metal bond lengths of these clusters. As shown in Figure S2 of the Supporting Information, the W–W bond lengths of W_3_ and W_5_ exceed by more than 1 Å the ones of bulk metallic W, indicating that W atoms behave more like single adatoms than compact clusters. The first coordination shell in the clusters roughly aligns with the second coordination shell in the bulk W, confirming the large atomic separation.

To investigate cluster oxidation, four O atoms were then added in the DFT calculations to each cluster, and the system was subjected to GO runs again. The resulting structures, reported in Figure 4b, show a general tendency for W atoms to be pulled away from the surface. This is particularly evident for W_1_: in W_1_O_4_, the metal atom is no longer embedded in the surface but adsorbed on top of an Fe_oct_ row. In W_3_O_4_, one of the three atoms remains embedded, while the other two adsorb on top of the second layer Fe_tet_ atoms, which represent the usual adsorption sites of metals on the reconstructed magnetite surface. For W_5_O_4_, three atoms adopt similar adsorption configurations, while one remains in the subsurface. Moreover, in all of the oxidized clusters, W–W distances increase further compared to the as-deposited counterparts. The pulling out of W atoms may suggest that the interaction with adsorbed O is stronger than the interaction with the lattice oxygen. The increased surface–W distance also explains the experimentally observed shift toward lower BE in W 4f spectra. These theoretical results demonstrate that clusters fragment spontaneously already upon deposition on the reconstructed surface and that the oxygen exposure further promotes this process. To understand the role of substrate defects introduced by the reconstruction, we also modeled pristine W clusters and their oxidized counterparts on an ideal bulk-terminated Fe_3_O_4_(001) surface.

Interestingly, on the non-reconstructed surface, W clusters retain their structures as clusters without fragmenting, as reported in the case of W_5_ in Figure 4b. Here, the average W–W coordination spans from 2 to 3, and all W atoms are also coordinated to Fe cations and few O atoms. The addition of only 4 O atoms to the cluster leads to a reorganization of W_5_ into a row-like geometry in which W atoms are connected to each other by O atoms. These results show that strong interactions between W and the reconstructed surface drive fragmentation upon deposition, while oxygen addition induces further disaggregation.

Mechanistically, the SCV-reconstructed Fe_3_O_4_(001) surface promotes fragmentation by providing a cation-deficient subsurface environment that facilitates the incorporation of incoming metal atoms, thereby circumventing the energy-demanding lattice reorganization required at an unreconstructed, fully coordinated surface. Given that some areas of the crystal may not be perfectly reconstructed, we expect that atomic species, fragments, and intact clusters coexist on the surface.

This is further supported by the calculations of the W 4f core levels. A comparison between computed core levels and experimental ones is reported in Figure 5a, as well as pristine W_1_ and W_5_ clusters core level spectra fitted using the theoretical core levels. On the SCV-reconstructed surface, W 4f_7/2_ core levels lie between 34.87 and 36.02 eV for all the clusters, independent of adsorption site, but only on the coordination with oxygen, i.e., larger coordination corresponds to higher core level binding energies (see Figure S3 in Supporting Information). The computed core levels correctly reproduce the shift toward lower BE that we find experimentally, confirming reduced substrate interaction for atoms adsorbed on top rather than embedded. Additionally, the disappearance of the W^6+^ spin–orbit doublet in W_1_ and its near-complete absence in W_3_ can be readily explained by the lack of tungsten–iron bonds following the cluster oxidation. Indeed, for a fixed coordination number of a tungsten atom, calculations show that its core-level binding energy increases when tungsten is coordinated not only to oxygen but also to iron atoms in the lattice. However, the computed core levels of W_5_ do not correctly reproduce the lower BE spectral doublet that we attributed to W^4+^ species. In contrast, W_5_ on the non-reconstructed surface (shown in Figure 4b) yields core levels (reported as gray triangles) that are about 2 eV lower than the main spectral doublet, but well describe the lower oxidation state component.

**Figure 5:**
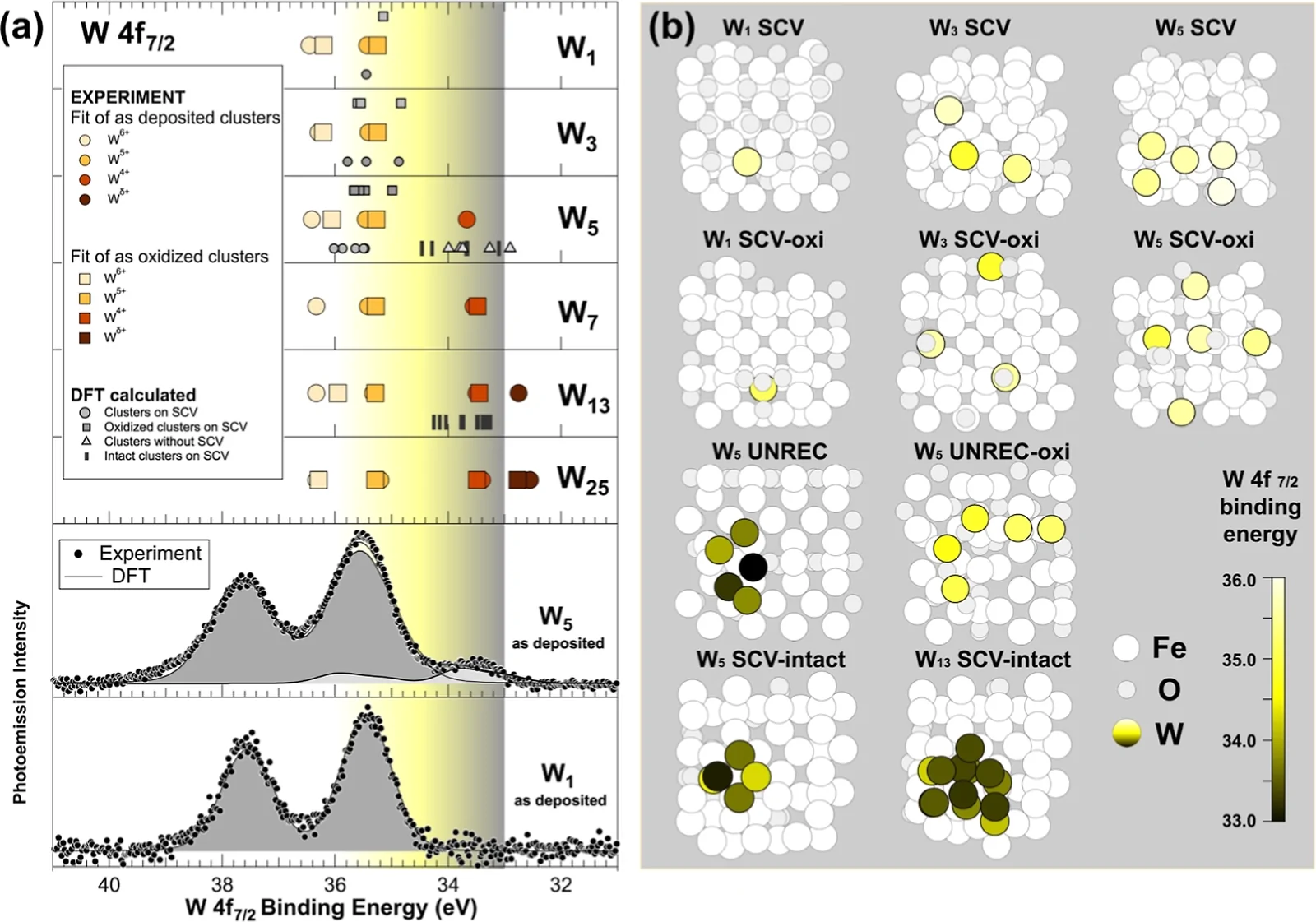
(a) In the upper panel, DFT-computed W 4f_7/2_ core levels for W atoms in W_1_, W_3_, and W_5_ (gray circles for as-deposited clusters, gray squares for oxidized ones, gray triangles for clusters on unreconstructed magnetite), as well as the BEs of fitting components of the experimental spectra of both as-deposited and oxidized clusters (in colors). For W_5_, the two families of W atoms correspond to clusters relaxed on the SCV-reconstructed surface (dark grey) and on the unreconstructed one (light grey). Black vertical rods indicate the core levels for the constrained intact W_5_ and W_13_ clusters shown in Figure 4c. In the lower panel, the fits of the experimental spectra (black dots) using DFT-computed core levels (gray curves) are reported. (b) Top-view color maps quantitatively showing the DFT-calculated 4f_7/2_ core electron binding energies for the W atoms of each investigated cluster configuration. Atoms belonging to the magnetite substrate layers are also included, with Fe atoms marked in white and O atoms in grey.

This again supports the coexistence of intact and fragmented clusters after deposition, consistent with the coexistence of reconstructed and non-reconstructed Fe_3_O_4_(001) areas on the experimental substrate. Additionally, upon introduction of oxygen, even the initially intact W_5_ fragments suggest that W–O interactions dominate over W–W and W–Fe interactions. Although DFT calculations have been performed only for the smaller clusters, we expect these conclusions to remain valid for larger clusters. W_13_ and W_25_ may be more resistant to complete fragmentation as the cohesive energy of a cluster increases with its size, yet the absence of any metallic component in the spectra indicates at least a partial breakup. Furthermore, upon oxygen exposure, fragmentation becomes dominant, as the main spectral components are those of W_1_ and W_3_, as we demonstrated for W_5_. To exclude the possibility that intact clusters could contribute to the observed high binding energy components corresponding to W^5+^ and W^6+^ species, we performed additional DFT calculations for W_5_ and W_13_ clusters supported on the SCV surface in non-fragmented configurations, obtained by placing the gas-phase global minima of the isolated species on the surface and subsequently relaxing the systems, whose local-minimum energy structures are shown in Figure 4c. In these constrained configurations, none of the W atoms reproduce the high-binding-energy components observed experimentally, as shown in Figure 5a with black vertical rods. These spectral features are recovered only when single atoms are allowed to form.

To quantitatively show this in a graphical way, in Figure 5b, top-view color maps of the DFT calculated 4f core electron binding energies for each W atom of every investigated cluster configuration are shown. The atoms of the magnetite substrate are also included, with Fe atoms marked with white and O atoms with gray. It can be noticed that for intact clusters, even W atoms at the interface with magnetite remain at a binding energy well-separated from that of single atoms, as colors are clearly shown according to the color scale also drawn behind the plots of Figure 5a.

Additional evidence of the ongoing breakup process comes from considering the integrated spectral area of each cluster as a function of oxygen exposure. While the area of W_1_, W_3_, and W_5_ remains constant throughout the oxidation, the area of the larger clusters increases by 7.8%, 8.3%, and 22.5% for, respectively, W_7_, W_13_, and W_25_. Since larger clusters are expected to show three-dimensional geometries, as already reported for W_13_ and W_25_ on graphene/Ru(0001), and the photoemission signal from atoms at the bottom of the clusters may be attenuated, the increase in spectral area implies a larger exposed surface area and therefore progressive fragmentation upon oxidation.

To understand whether similar fragmentation behavior also occurs for other early transition metal nanoclusters, we repeated the experiment using atomically precise Mo_n_ (*n* = 1, 5, 13) and Ta_n_ (*n* = 1, 5, 25) nanoclusters. Mo 3d and Ta 4f core level spectra of pristine clusters deposited at 300 K on magnetite are reported in Figure 6. The Mo single atom spectrum (see Figure 6c) was fitted using two spin–orbit doublets at 231.4–234.5 eV and 232.18–235.28 eV, corresponding to Mo^5+^ and Mo^6+^, respectively. For Mo_5_ and Mo_13_, an additional component, assigned to Mo atoms in the 4+ oxidation state for Mo_13_ and to an intermediate oxidation state δ+ for Mo_5_, was required, with the 3d_5/2_ component in the range of 230.3–231.3 eV.

**Figure 6:**
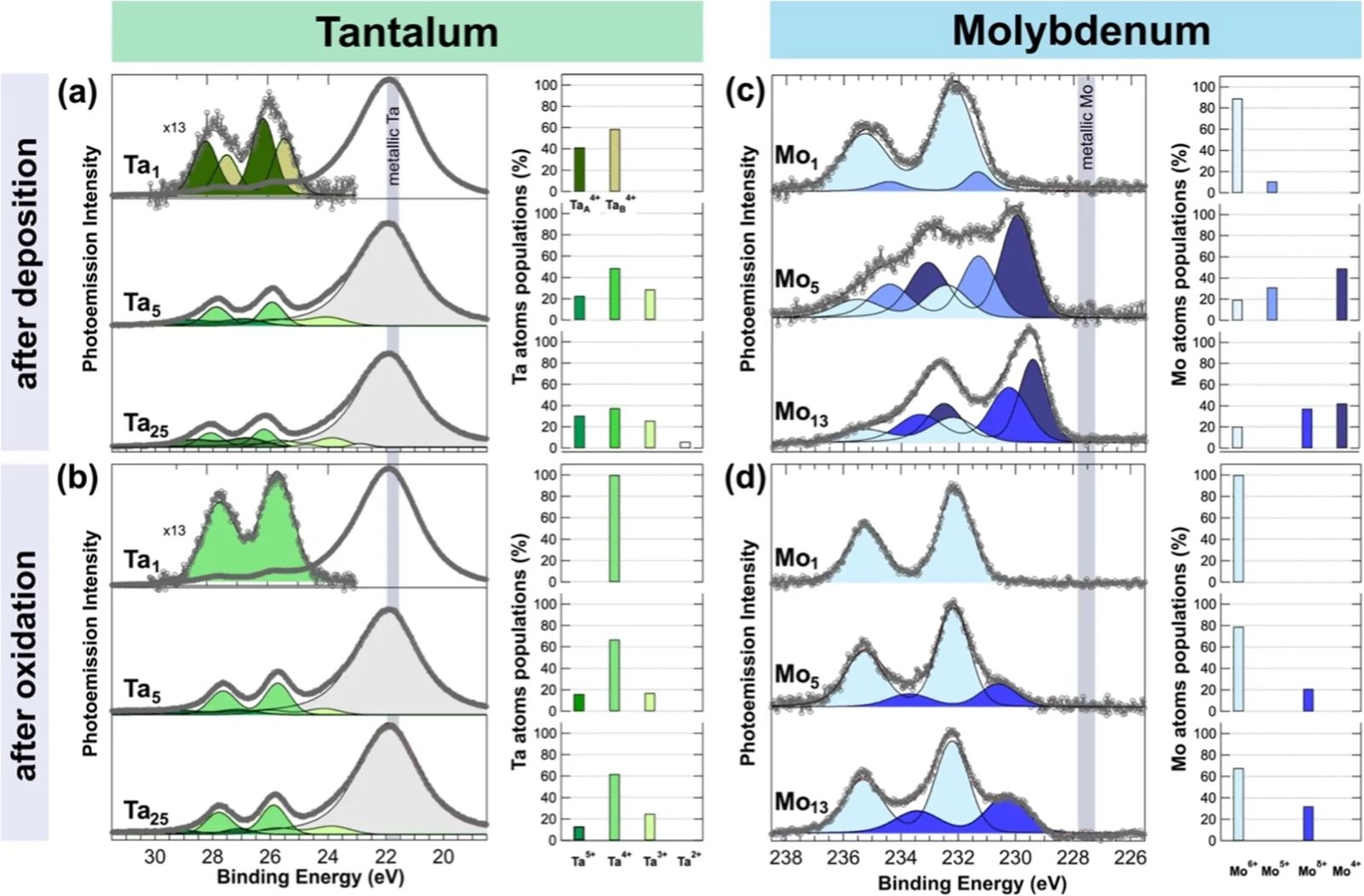
(a, b) Fitted Ta 4f core level spectra of Ta_1_, Ta_5_, and Ta_25_ nanoclusters (a) after deposition and (b) after oxygen exposure. Given the low coverage of deposited Ta monomers, the Ta_1_ spectrum is also shown after background subtraction and with intensity scaling to make the spectral components more readily visible. Different colors are used to identify various spin orbit doublets attributed to different oxidation states. The same colors are used on the left for the bars of the histograms of the Ta atomic populations as a function of Ta-oxidation state. The component related to the O 2s core level around 22 eV is highlighted in gray, considered as the background. (c, d) Fitted Mo 3d core level spectra of Mo_1_, Mo_5_, and Mo_13_ nanoclusters (c) after deposition and (d) after oxygen exposure. Different colors are used to identify various spin orbit doublets attributed to different oxidation states. The same colors are used on the left for the bars of the histograms of the Mo atomic populations as a function of Mo-oxidation state.

Unlike W nanoclusters, where the W^5+^ oxidation state dominates in monomers, the 5+ oxidation in Mo monomers represents only a minor fraction of the spectral weight, as shown in Figure 6c, and gains significant intensity only in larger clusters. At a first glance, the Ta core level spectra appear similar across all cluster sizes, displaying a single, well-defined spin–orbit doublet. However, fitting reveals that an increasing number of spectral components is required as the cluster size grows. Ta_1_ can be fitted using two closely spaced components above 25 eV, within the energy range characteristic of Ta^4+^. For Ta_5_, additional Ta^3+^ and Ta^5+^ components are needed, located at 24.05 and 26.91 eV, respectively. Ta_25_ requires yet another component, assigned to Ta^2+^, at 22.9 eV. Notably, and unlike W clusters, even as-deposited Ta_25_ is dominated by higher-oxidation-state components (see Figure 6a), suggesting a greater degree of fragmentation.

Cluster oxidation was performed by exposing the sample to oxygen within the same pressure range used for W clusters, with total exposure of 436 and 530 L for Ta and Mo, respectively. The corresponding post-oxidation core-level spectra are shown in Figures 6b and d.

The most pronounced changes occur in the Mo clusters, where, notably, no significant binding-energy shifts are observed among the different components. The key difference is the disappearance of the Mo^4+^ component in both Mo_5_ and Mo_13_, as also evident from the histograms in Figure 6d, along with the emergence of the Mo^δ+^ component in Mo_5_. In contrast, the Ta 4f core-level spectra show only minor modifications, primarily a slight shift to lower binding energies. Detailed analysis, however, reveals an increase in the Ta^4+^ component at the expense of the Ta^2+^ and Ta^3+^ components in both Ta_5_ and Ta_25_, while in Ta_1_, only one of the two initial components remains. The absence of metallic contributions in both Mo and Ta clusters strongly supports the conclusion that these systems also undergo fragmentation, as metallic components would be expected for large clusters due to their larger similarity to bulk metal. Nevertheless, the strong similarity of the Ta 4f spectra across all cluster sizes may indicate that Ta clusters fragment even more extensively, yielding small aggregates that are largely independent of their initial size.

A comparison of the integrated area of the Mo 3d core-level spectra before and after the oxidation shows an increase of Mo_5_ and Mo_13_ areas of +20% and +4%, respectively, consistent with this interpretation, while the area of Mo_1_ remains unchanged. In contrast, the spectral area of all Ta clusters deviates by a maximum of 1.5% before and after oxidation, indicating a significantly larger degree of fragmentation of Ta clusters occurring already after deposition.

DFT calculations reveal that Ta and Mo exhibit a propensity for oxidation on the reconstructed Fe_3_O_4_ termination comparable to that of W. In all investigated cases, namely, Mo and Ta species with numbers of atoms of 1 and 5, respectively, oxidation induces significant local surface disorder, characterized by the formation of under-coordinated Fe atoms (see Figure S4), ultimately leading to fragmentation of the supported clusters (see Figure S4 in the Supporting Information).

Analysis of the global minimum structures indicates that Mo and Ta atoms preferentially occupy the characteristic Fe_oct_-Fe_3_O_4_ surface adsorption site located between the octahedral Fe rows and the adjacent rows of tetrahedrally coordinated Fe atoms. This is an interesting difference from W_1_, which prefers to be incorporated into the magnetite lattice. Accommodation of Ta within this site enables the formation of a quasi-octahedral oxygen coordination, which, in turn, induces a substantial distortion of the octahedral Fe rows. In particular, some of the uppermost Fe_oct_ atoms are displaced as they attempt to recover the lost oxygen coordination by bonding to the nearest Fe_tet_. In the case of single-atom Mo deposition, the displacement of the Fe_oct_ rows is less pronounced, consistent with Mo adopting a predominantly pentahedral-like oxygen coordination with fewer oxygen neighbors while also interacting with two underlying Fe atoms. This is consistent with the larger atomic radius of Ta, which protrudes from the surface in such a way that it can bind a surface oxygen in the form of a Ta=O species.

For larger deposited species such as Ta_5_ and Mo_5_, the global-minimum structures confirm a pronounced distortion of the Fe_3_O_4_ surface, with Ta_5_ closely resembling W_5_ in its adsorption pattern. (see Figure S4 in the Supporting Information). A key difference among the deposited Mo, Ta, and W species emerges from the neighbor analysis, which reveals a markedly stronger affinity of Mo for Fe. Specifically, in the Mo_5_ system, four out of the five deposited Mo atoms, each coordinated to four oxygen atoms, also have one or two Fe neighbors at short distances (2.5–2.8 Å). This close metal–metal proximity has a significant impact on the oxidation states of the deposited atoms, as evidenced by Bader charge analysis (Figure S5 in Supporting Information), with Ta and W atoms derived from Ta_5_ and W_5_ clusters exhibiting nearly uniform charges within ±3%, whereas Mo atoms displaying a much broader charge distribution, with deviations reaching up to ∼18%.

A key characteristic of all these systems is the spontaneous fragmentation, partial or complete, of the clusters upon deposition. This behavior results from a competition between metal–metal and metal–oxygen interactions, including those involving the support. If one considers bulk properties, such as the oxide formation enthalpy normalized per mole of oxygen atoms,^41^ a clear trend emerges for the tendency of Ta, W, and Mo to form oxides, which is in good agreement with the experimental observations. Indeed, as reported in Table 1, where the values for the most stable oxides are summarized, the formation enthalpies of W and Mo oxides are only slightly higher than that of iron oxide, whereas the value for tantalum oxide is significantly larger.

**Table 1:** Thermodynamic Properties of Ta, W, Mo, and Fe Oxides; Formation Enthalpies of the Most Stable Oxide of Each Metal Are Reported Per Mole of Oxygen Atoms (kJ mol^–1^ O) in Column 1, alongside the Corresponding Oxophilicity Values (kJ mol^–1^) in Column 2, Calculated as the Difference between the Heat of Sublimation of the Solid Metal and the Heat of Formation of Its Most Stable Oxide Per Mole of Metal Atoms; Thermodynamic Data Were Obtained from the NIST-JANAF Thermochemical Tables Database^42^

metal	most stable oxide	normalized enthalpy per mole of O atoms (kJ/mol O)	calculated oxophilicity (kJ/mol)
tantalum	Ta_2_O_5_	–409.2	1805
tungsten	WO_2_	–295	1441
molybdenum	MoO_2_	–294	1439
iron	Fe_3_O_4_	–280.3	790

On the other hand, when considering oxophilicity, defined as the heat of sublimation of M­(solid) minus the heat of formation of the most stable oxide of metal M (from M­(solid) + *O*_2_(*g*)), normalized per mole of metal atoms,^41^ this parameter predicts a clear trend: Ta > W > Mo ≫ Fe. This order is consistent with the experimentally observed relative tendency toward cluster fragmentation and oxidation.

A similar approach but centered on metal–support interactions for single atoms and nanoparticles of several transition metals deposited on different metal oxides, including magnetite, has been recently employed by Wang et al.^26^ who identified the adhesion energy as a useful descriptor for rationalizing differences among metals. This energy contains two main contributions: the metal–oxygen interaction term Q­(MO), and the metal–metal interaction term between the adsorbate (M) and the substrate metal (M′), Q­(MM′). The Q­(MO) term reflects the oxophilicity of metal M, as reported in ref 41, whereas Q­(MM′) represents the M–M′ affinity derived from the formation enthalpy of M–M′ alloys. In this model, the adhesion energy also includes a coefficient proportional to the electronegativity ratio between the substrate metal and the adsorbed metal, χ­(M′)/χ­(M). It has been shown that, across a period of the transition metals, Q­(MO) decreases, reaching its minimum for coinage metals, while Q­(MM′) increases significantly. This trend indicates that M–M′ interactions become progressively more important for late transition metals. Although W, Mo, and Ta were not explicitly examined in that study, useful insights can be obtained by applying the same concepts. Computing the χ­(M′)/χ­(M) ratio for Fe and the supported metals yields values of 1.22 for Ta, 0.78 for W, and 0.85 for Mo. Because this ratio reflects the competing bonding affinity of M′ and M, larger values indicate a stronger tendency to form interfacial M–O bonds. Accordingly, Ta clusters should be more prone to form metal–oxygen bonds at the interface than W and Mo, consistent with the more extensive fragmentation observed for Ta nanoclusters. Indeed, although theoretical calculations on small clusters predict fragmentation regardless of the metal species, a key difference emerges in the XPS spectra of larger clusters. In the case of W, clear distinctions are observed between the W_25_ core level spectrum and those of W_1_ or W_5_. While the absence of a metallic component has been attributed to cluster fragmentation, this mechanism appears to be only partial for W, as the spectrum of the largest cluster still shows dominant contributions from lower oxidation states. In contrast, core level spectra of Ta_5_ and Ta_25_ are remarkably similar: their superposition reveals only a shift towards lower BEs of the main spectral components. This distinctive behavior points to a more extensive fragmentation of Ta clusters, persisting even at larger cluster sizes. This interpretation is further supported by the trend in metal–metal bond dissociation energies, reported to be 5, 4.5, and 4 eV for W, Mo, and Ta, respectively,^43^ indicating that Ta–Ta bonds are the weakest and therefore most easily disrupted.

However, electronegativity ratios alone cannot fully account for the cluster stability. Pt clusters, for example, are known to remain intact on Fe_3_O_4_(001) and exhibit an electronegativity ratio of 0.8, like those of W and Mo. Thus, fragmentation cannot be explained solely based on this coefficient.

Indeed, Wang et al.^26^ demonstrated that the Q­(MM′)/Q­(MO) ratio increases across a transition-metal period, implying that M–M′ interactions dominate for late transition metals and that the adhesion energy decreases correspondingly. This framework explains why Pt clusters, which preferentially form Pt–Fe rather than Pt–O bonds at the interface, remain stable, whereas W and Mo clusters can fragment due to the favored formation of metal–oxygen bonds.

The strong tendency of these metals to form M–O bonds also helps to rationalize why W atoms replace Fe atoms in the magnetite lattice. Similar Fe-cation substitution was observed by Sharp et al.^29^ for Rh atoms, although only after annealing the system to 720 K. The higher oxygen affinity of W is reflected in the enthalpies of formation of their highest-oxidation-state oxides: WO_3_ (−842.7 kJ/mol)^44^ versus RhO_2_ (−224.9 kJ/mol).^45^ The substantially stronger W–O bonding likely compensates for the energetic cost associated with cation displacement.

Metal–substrate interaction strength can also be described through the adsorption energy of a metal atom on the substrate. Wang et al.^26^ showed that the adsorption energy scales with Q­(MO), Q­(MM′), or a linear combination of both. Because W, Mo, and Ta interact strongly with oxygen, one expects high adsorption energies for these metals on Fe_3_O_4_(001), which would enhance their propensity to fragment. Cao et al.^46^ further correlated the fragmentation tendency of oxide nanoparticles on TiO_2_ with their adsorption energy: the higher the adsorption energy, the more readily the nanoparticles fragment. This relationship fully aligns with the observed behavior of W, Mo, and Ta nanoclusters. A direct computational determination of the adsorption energies of Ta_1_, W_1_, and Mo_1_ on Fe_3_O_4_ provides additional and more quantitative validation of this interpretation: the trend in the adsorption energies of single atoms, evaluated at the DFT–PBE level with respect to isolated gas-phase atoms as a reference, increases from Mo (8.4 eV) to W (11.2 eV) and Ta (13.9 eV).

Furthermore, we highlight that the incorporation of oxidized metal atoms into the SCV-reconstructed Fe_3_O_4_(001) surface, accompanied by Fe-cation displacement and substitution, is a feasible process at room temperature. It is driven by the energy released upon oxidation and facilitated by the cation-deficient subsurface structure, which lowers the energetic cost of the Fe displacement and lattice reorganization. Within a TST framework, the energy released during oxidation is estimated to generate a transient local temperature increase sufficient to make incorporation barriers in the range of ca. 1.6–2.0 eV accessible on a one-second time scale (assuming a typical vibrational prefactor of 10^13^ s^–1^). The barrier range inferred from our TST analysis is consistent with the activation-energy scale of approximately 1.3–1.4 eV estimated from the annealing data provided by ref 38. This interpretation is consistent with previous studies showing that incorporation of strongly oxophilic metals like Ti and Co into the SCV reconstruction is energetically preferred over simple surface adsorption.^38^ The correlation between oxide formation enthalpies and incorporation propensity further supports metal–oxygen bond formation as the thermodynamic driving force for incorporation.

Finally, we note that the fragmentation of W, Ta, and Mo clusters is not driven by high temperature. The Hüttig temperatures of these metals, i.e., the temperatures at which surface atom mobility becomes significant, are all well above 300 K. This suggests that the observed fragmentation is influenced by the interactions between the clusters and the substrate, rather than by thermal effects.

## Conclusions

In summary, we have shown that early transition-metal (W, Mo, and Ta) nanoclusters with precise nuclearity undergo spontaneous, adsorption-induced fragmentation upon deposition on reconstructed Fe_3_O_4_(001) at room temperature. The dissociation process takes place in the absence of high temperature or reactive environments. HR-XPS analysis reveals the systematic and consistent absence of metallic spectral components and the dominance of highly oxidized species even for the large clusters formed by 13 and 25 atoms. The experimental findings are supported by DFT calculations showing that strong metal–oxygen interactions and surface reconstruction–induced defects in magnetite drive the partial dissociation of clusters into monomers or small aggregates. Further oxygen exposure promotes the single-atom formation, particularly for larger clusters, confirming that M–O bonding dominates over metal–metal cohesion in these systems. The fragmentation propensity follows the trend Ta > W > Mo and correlates with calculated adsorption energies, oxophilicity, and metal–metal bond strengths, highlighting the decisive role of metal–support interactions rather than cluster size or temperature. Our findings establish cluster fragmentation as an intrinsic stabilization pathway for early transition metals on reducible oxides and provide a mechanistic framework for deliberately generating high densities of atoms under room temperature reaction conditions, with direct implications for the design of novel heterogeneous catalysts.

## Experimental Methods

### Substrate Preparation

The Fe_3_O_4_(001) single crystal was cleaned in the preparation chamber by several cycles of Ar^+^ sputtering at an Argon pressure of 3 × 10^–6^ mbar. This was performed using 1 keV Ar^+^ ions incident normally to the surface for a duration of 90 min. After sputtering, the sample was annealed at 900 K for 20 min in an oxygen atmosphere (*p* = 1 × 10^–6^ mbar) to restore surface stoichiometry. The long-range crystalline order and the $\left(\sqrt{2}\times \sqrt{2}\right)$ R45° surface reconstruction, consistent with the SCV model, were verified by LEED. To ensure a deeper structural characterization, a LEED intensity versus voltage (LEED-IV) analysis was performed, confirming the high quality and reproducibility of the substrate surface. More specifically, diffraction patterns were acquired with electron energies between 40 and 400 eV with a step of 2 eV. From these, intensity curves as a function of energy were extracted and averaged over equivalent spots. In this way, 30 IV curves were obtained for symmetrically inequivalent diffraction orders, and they were compared with those reported by Parkinson et al.,^47^ yielding an average Pendry R factor of 0.16 (see Figure S6). Surface cleanliness and absence of contaminants was checked by HR-XPS. Real-space morphology of the clean $\left(\sqrt{2}\times \sqrt{2}\right)$ R45° surface and after W atom deposition was investigated by STM on the CoSMoS branch line facility. STM topographs were acquired at room temperature using Aarhus STMs equipped with a PtIr tip. Image processing and analysis were performed with the WSxM software package.^48^

### Cluster Generation and Deposition

Cationic nanoclusters of tungsten (W_*n*_^+^, *n* = 3, 5, 7, 13, 25), molybdenum (Mo_*n*_^+^, *n* = 1, 5, 13), and tantalum (Ta_*n*_^+^, *n* = 1, 5, 25) were produced using the ENAC (Exact Number of Atoms in each Cluster) laser ablation source. The clusters were generated by ablating a rotating metal (W, Ta, or Mo) target with a Nd:YAG laser (l = 532 nm) and mass-selected by a quadrupole mass filter (QMS).^49–53^ Mass selection of different clusters was monitored by acquiring mass spectra with the QMS (Figure S1). Clusters were deposited in situ onto the Fe_3_O_4_(001) surface in soft-landing conditions, with a kinetic energy maintained below 1 eV per atom to prevent fragmentation upon impact.^54^ The substrate was kept at room temperature during deposition. The amount of deposited clusters was controlled by integrating the cluster current on the sample over time.

For the deposition of tungsten and molybdenum monomers (W_1_), dedicated high-purity tungsten and molybdenum wire evaporators were used. The evaporators were attached directly to the SuperESCA chamber, allowing for the simultaneous measurements of the XPS spectra and determination of coverage. Ta monomers were instead deposited by means of ENAC as larger clusters.

### Oxygen Exposure

After deposition, the clusters were exposed to molecular oxygen to investigate their oxygen uptake mechanism. More specifically, W and Ta clusters were exposed to a pressure of 7 × 10^–10^ mbar for approximately 150 s, then in sequence the pressure was increased to 1 × 10^–9^ mbar for 270 s, 5 × 10^–9^ mbar for 390 s, 1 × 10^–8^ mbar for 390 s, 5 × 10^–8^ mbar for 600 s, 1 × 10^–7^ mbar for 330 s, 5 × 10^–7^ mbar for 300 s, and finally 1 × 10^–6^ mbar for 360 s, with a final exposure of 436 L. Mo clusters were exposed to molecular oxygen in the same pressure ranges, for a total exposure of 535 L. This procedure was adopted in order to follow the oxygen uptake, which is typically fast at the beginning, thanks to the large availability of dissociation sites, and then slows down when one approaches saturation. This is the reason why in the first step the pressure was kept at its lowest value in order for exposure to increase as slowly as possible, thus following the critical initial phase by means of fast XPS acquisition. In the following steps, pressure was increased whenever no change was detected between one fast spectrum and the following, gradually making exposure change faster until saturation was reached.

### HR-XPS Measurements

HR-XPS measurements were performed at the SuperESCA beamline of Elettra.^55,56^ In order to optimize surface sensitivity and signal, all W 4f core-level spectra were acquired at normal emission and with a photon energy of 200 eV. This energy was specifically chosen to maximize the ratio between the photoionization cross sections of the W 4f core levels and the O 2s and W 5p ones, which would otherwise influence the background. Similar considerations were applied to Mo 3d (320 eV of incident photon energy) and Ta 4f (200 eV). The overall energy resolution was better than 50 meV (W and Ta) and 70 meV (Mo). The BE scale was calibrated by acquiring the Fermi level of a clean Au foil in electrical contact with the sample of interest, ensuring an absolute and reproducible energy reference. To follow in real time the oxygen uptake spectra, fast XPS spectra were acquired in the same way but with a shorter acquisition time per spectrum. Indeed, this parameter was equal to 80 s for high-resolution spectra, whereas it was of only 29 s for the fast ones. Almost 100 spectra were acquired for each oxygen uptake.

### Data Analysis

The photoemission spectra were analyzed through a multi-component fit using a Doniach-Šunjić line shape^57^ convoluted with a Gaussian, with an asymmetry parameter set to zero as non-metallic components were never observed. The choice of Gaussian and Lorentzian widths was optimized to account for both the core-hole lifetime and the dominant contribution of vibrational and inhomogeneous broadening. Specifically, the Lorentzian width was chosen according to literature,^58^ keeping the 4f_7/2_ peak lower than that of the 4f_5/2_ component, in accordance with the existence of Koster–Kronig transitions. The Gaussian widths were determined through the fit to reflect the local chemical environment’s heterogeneity too.

For the background subtraction of W 4f core level spectra, a two-step strategy was adopted to isolate the cluster signal from the complex substrate background. The spectral region chosen for analysis included the Fe 3p and O 2s peaks, as these substrate features overlap with the binding energy region of the metal clusters’ core levels. A background spectrum of the bare Fe_3_O_4_(001) substrate was acquired before deposition of each cluster. This was fitted with a polynomial function and used to model the background. The same procedures were adopted to analyze the fast XPS spectra. For Mo 3d and Ta 4f core level spectra, the background was modeled using a polynomial function.

## Theoretical Methods

In the absence of direct experimental structural information about the deposited clusters on the Fe_3_O_4_ surface, we resorted to a stochastic structure-prediction protocol to identify energetically favorable structural models. Specifically, we used a DF-GO approach,^40,59^ a first-principles version of the Basin Hopping (BH) algorithm,^40^ as implemented in an in-house Python-based code.

The BH procedure starts from an initial configuration, arbitrarily or randomly chosen, defined by a set of 3N atomic coordinates, where N corresponds to the nuclearity of the system under consideration. New configurations are generated by applying random perturbations to a selected subset *S* of these atomic coordinates, thereby allowing the inclusion of possible constraints on the system, followed by local geometry optimizations. Each new local minimum is accepted or rejected according to a Metropolis criterion, with acceptance probability *P* = min­(1,exp­(−Δ*E*/*T*)), where Δ*E* is the energy difference between consecutive minima and *T* is a fictitious algorithmic temperature used to control the acceptance of energetically uphill moves. The algorithm iteratively proceeds by generating and sampling new local minima starting from the currently accepted configuration and is eventually terminated when no new structures with energies lower than the current best minimum are identified over a number of consecutive basin-hopping steps.

We employed square-cell slab models to represent both the reconstructed and unreconstructed Fe_3_O_4_(001) terminations. The reconstructed surface consisted of 7 layers (*N* = 106 atoms) with a lattice parameter of 11.87 Å. Five surface layers (3 in the unrestructured case) were allowed to fully relax. The energy evolution as a function of the basin-hopping iteration for a representative Fe_3_O_4_/W system is reported in Figure S7.

GO runs were carried out using DFT energies evaluated at the Perdew–Burke–Ernzerhof (PBE) exchange–correlation functional level,^60^ as implemented in the OPENMX code.^61^ OPENMX solves the Kohn–Sham equations within a pseudopotential-based linear combination of numerical atomic orbitals framework under periodic boundary conditions. GO runs were performed within a spin-unrestricted-DFT framework initialized from a spin arrangement inspired by the bulk Fe_3_O_4_ ferrimagnetic pattern and allowed to relax during the stochastic search. In all calculations, optimized numerical pseudo-atomic basis sets of the “Quick” type^62^ were employed together with norm-conserving PBE pseudopotentials.^63^ An energy cutoff of 150 Ry was used to define the real-space grid employed to evaluate the Hartree potential using an FFT-based Poisson solver.^64^ Owing to the large surface supercell employed, Brillouin-zone sampling was restricted to the sole Gamma point.

XPS core-level binding energies of the W 4f states were calculated for the putative global-minimum structures using the all-electron FLEUR code,^65^ which implements the full-potential linearized augmented plane-wave method.^66^ Within this approach, space is partitioned into two regions: muffin-tin spheres centered on the nuclei and the remaining interstitial region. Valence states, which extend throughout the entire simulation cell, are represented using the LAPW basis, a hybrid representation consisting of plane waves in the interstitial region and localized atom-centered functions inside the muffin-tin spheres.

Core electrons are treated separately by solving the fully relativistic Kohn–Sham–Dirac equations on a radial grid inside the muffin-tin spheres, using the spherical component of the effective potential obtained self-consistently from the total electron density. The core-electron wavefunctions are smoothly extended beyond the muffin-tin boundary, and the resulting exponentially decaying core-tail charge density is consistently included in the total electron density in the interstitial region during the self-consistent cycle. This methodology enables an accurate and physically sound description of core-level binding energies and relativistic multiplet effects for all atomic species.

The LAPW basis was defined by a plane-wave cutoff of *K*_max_ = 5.0 Å^–1^ for the interstitial region and by atom-specific muffin-tin radii, chosen to avoid sphere overlap. Inside the muffin-tin spheres, spherical harmonics up to *l*_max_ = 8 were employed. Local orbitals were added for the W 5s and 5p semicore states and for the Fe 3s and 3p states to improve their description and reduce linearization errors. The surface systems were modeled using two-dimensional periodic boundary conditions, and Brillouin-zone integrations were performed using a Γ-centered 2 × 2k-point mesh in the surface plane.

## Supplementary Material



## Notes

Mass spectrum of W^+^, Ta^+^, and Mo^+^ nanoclusters; histograms of W–W distance distribution; computed W 4f_7/2_ core levels as a function of W–O coordination; theoretical models of Ta_1_, Ta_5_, Mo_1_, and Mo_5_ clusters supported on reconstructed Fe_3_O_4_(001); computed Bader charge W_5_, Mo_5_, and Ta_5_ atoms as a function of average metal–oxygen bond lengths; computed LEED-IV curves; and energy evolution as a function of basin-hopping iteration for a representative Fe_3_O_4_/W system (PDF)
