# Supplementary material for: Room-Temperature Adsorption on Magnetite Induces Spontaneous Fragmentation of Early Transition-Metal Size-Selected Nanoclusters
Source: J Am Chem Soc. 2026 Sep 17;148(38):41612–25. doi: 10.1021/jacs.6c15419 (PMC13632546; doi:10.1021/jacs.6c15419)
Supplement: Supplementary file 1 [file ja6c15419_si_001.pdf]

Supporting Information: Room Temperature Adsorption on Magnetite Induces Fragmentation of Early Transition-Metal Size-Selected Nanoclusters

Gabriele Coltrioli[1,#], Deborah Perco[1,#], Luca Sementa[2,#], Marco Bianchi[3], Andrea Berti[1], Mikhail Bandurist[1], Paolo Lacovig[3], Silvano Lizzit[3], Aras Kartouzian[3], Ueli Heiz[4], Alessandro Fortunelli[5] and Alessandro Baraldi[1,3,*]

[1] Department of Physics, University of Trieste, Via Valerio 2, 34127 Trieste, Italy.

[2] Istituto per i Processi Chimico-Fisici, Consiglio Nazionale delle Ricerche, Via Giovanni Moruzzi 1, Pisa 56124, Italy.

[3] Elettra - Sincrotrone Trieste, S.S. 14 Km 163.5, AREA Science Park, 34149 Basovizza-Trieste, Italy.

[4] Chemistry Department & Catalysis Research Center, Technical University of Munich, Lichtenbergstrasse 4, Garching D-85748, Germany.

[5] Istituto di Chimica dei Composti Organometallici, Consiglio Nazionale delle Ricerche (CNR-ICCOM), Via Giovanni Moruzzi 1, 56124 Pisa, Italy.

KEYWORDS: fragmentation, oxidation, clusters, XPS, STM, DFT, metal-substrate interaction

*Corresponding Author: alessandro.baraldi@elettra.eu

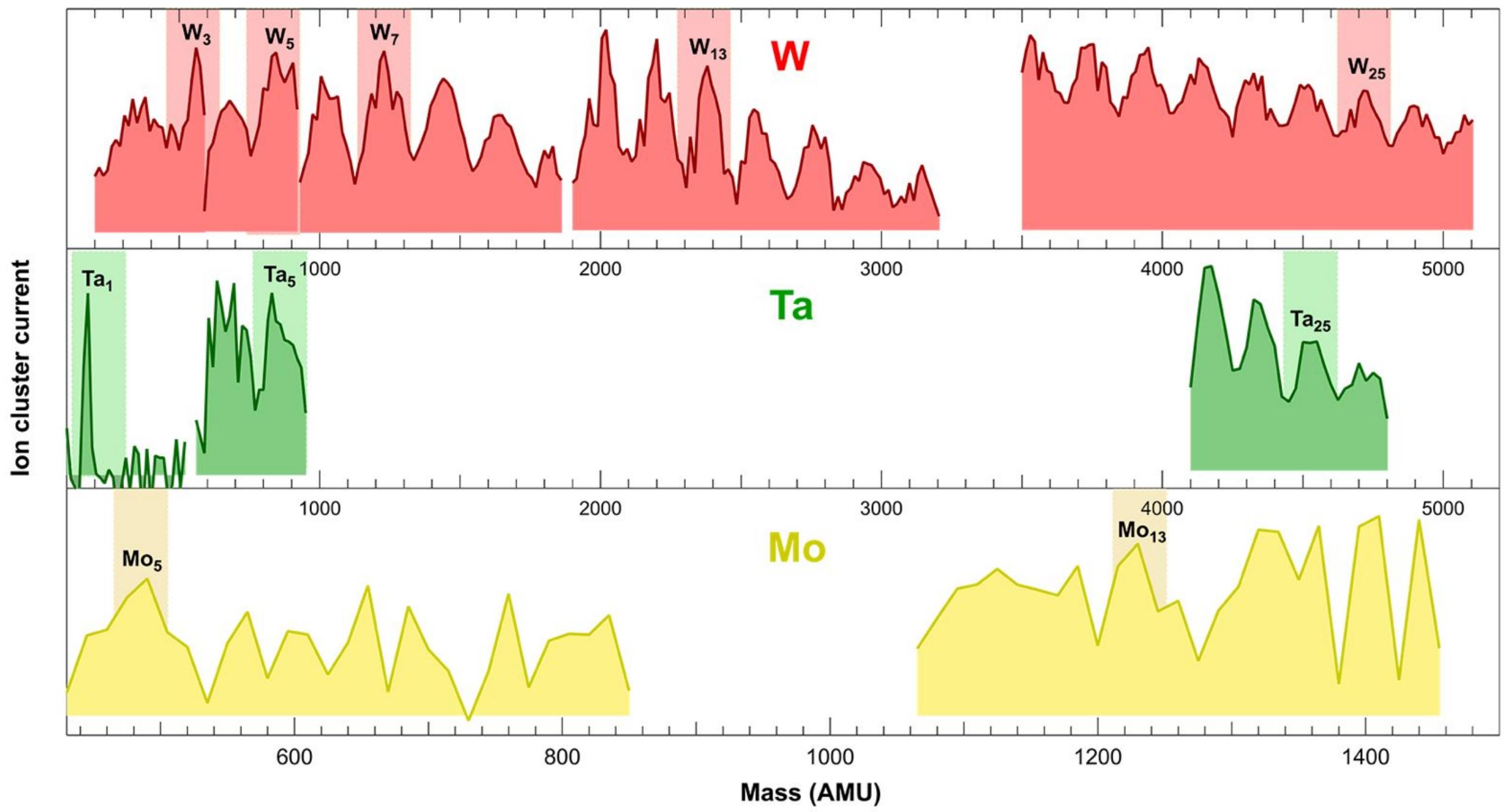

Figure 1S. Mass spectra of W_n^+, Ta_n^+ and Mo_n^+ clusters generated by ENAC.

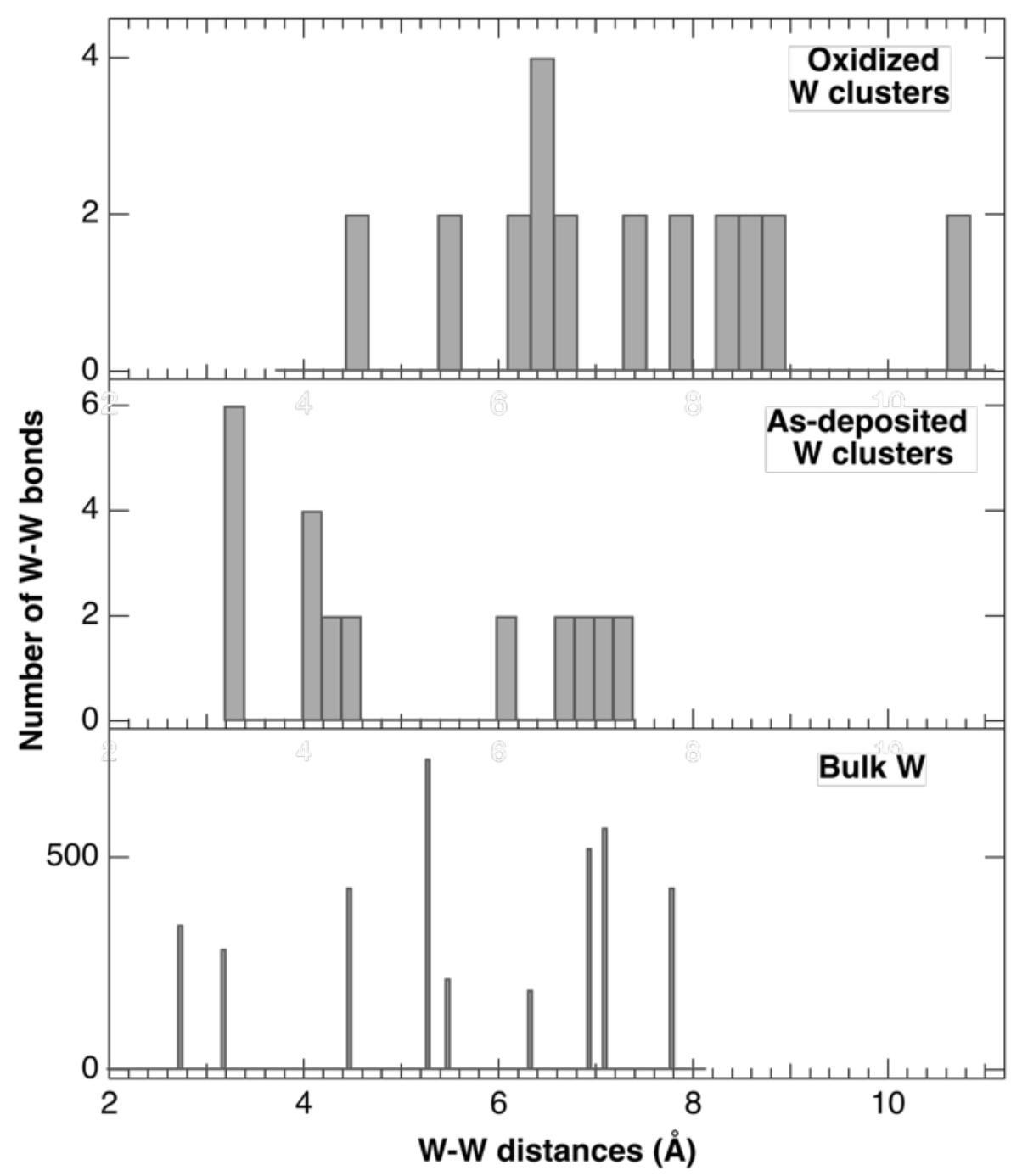

Figure 2S. Comparison of W-W distance distribution in as-deposited and oxidized nanoclusters, and in bulk W.

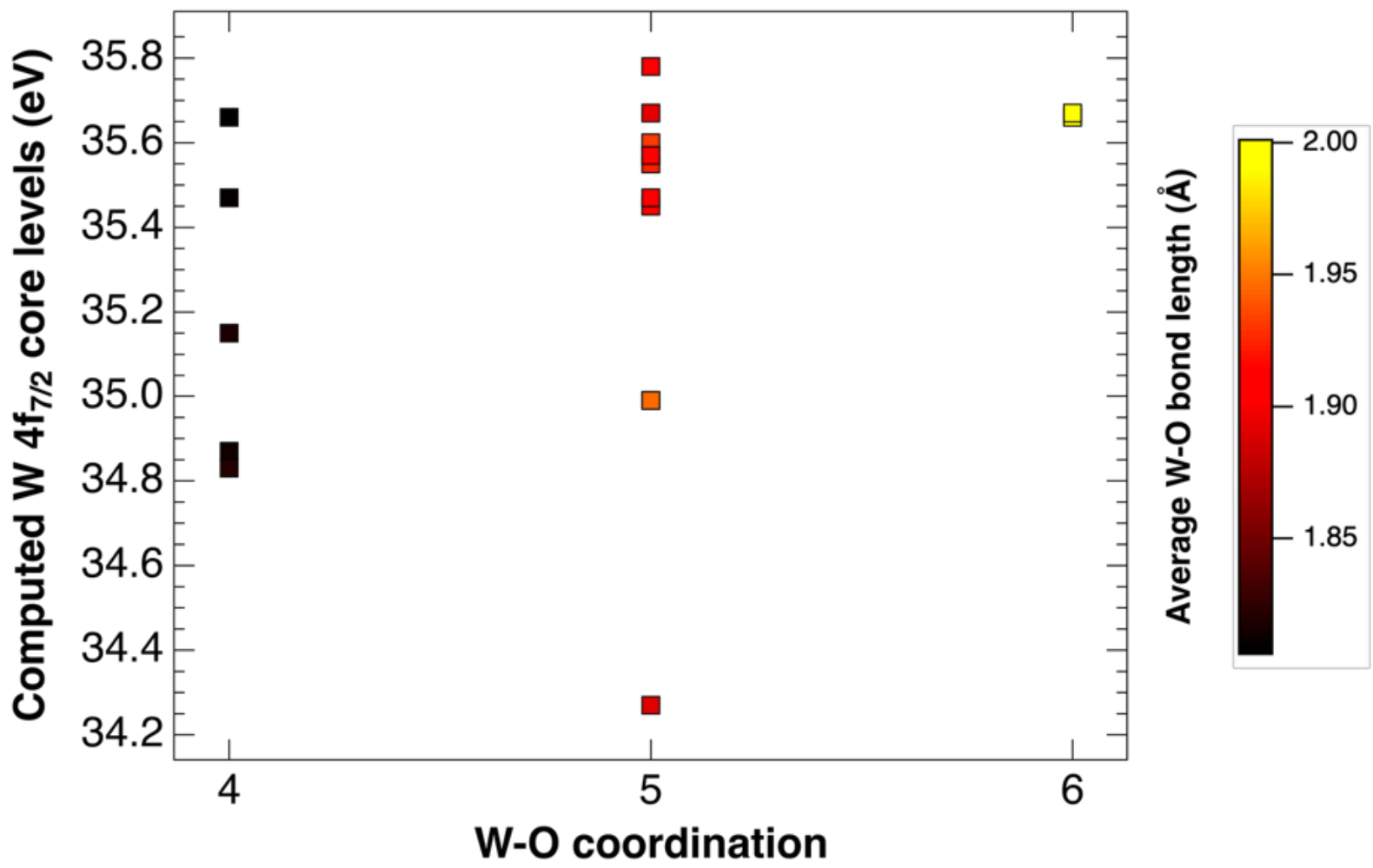

Figure 3S. Computed W $4f_{7/2}$ core levels as a function of the W-O coordination, for W clusters on SCV-reconstructed surface. The color scale represents the average W-O bond length distribution.

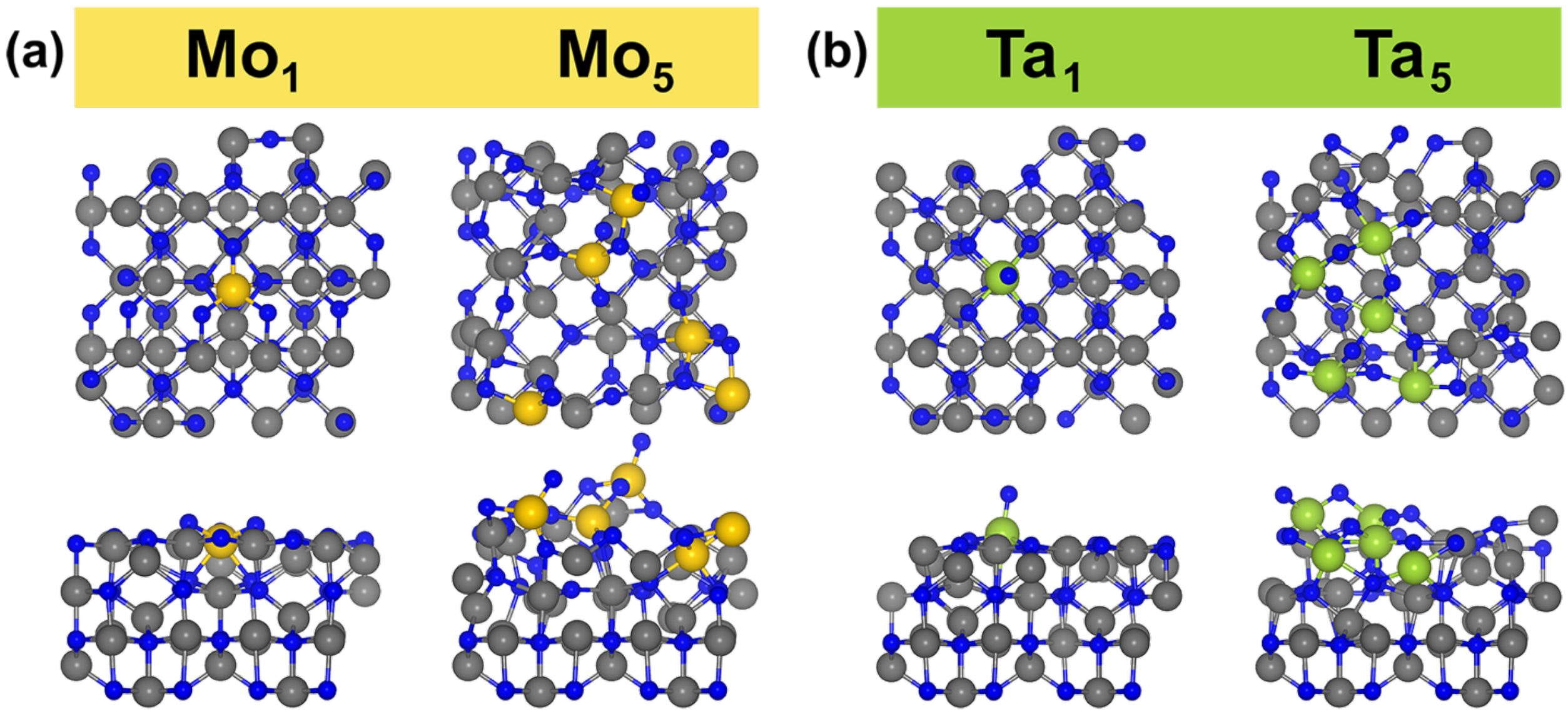

Figure 4S. Top and side views of the DFT@GO-optimized configurations of as-deposited Mo_1, Mo_5, Ta_1 and Ta_5 on Subsurface Vacant Cation model reconstructed $Fe_3O_4(001)$.

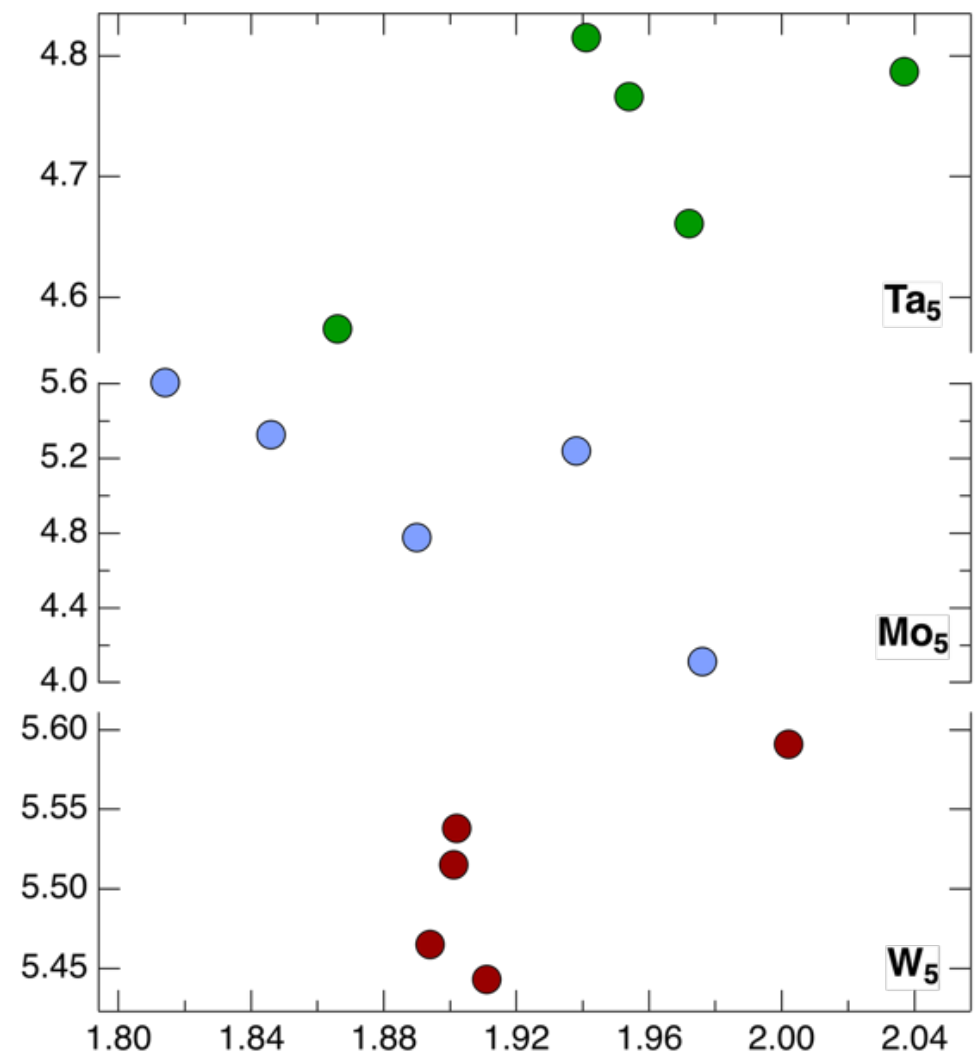

Figure 5S. Computed Bader charge of $Fe_3O_4(001)$-supported W_5, Mo_5 and Ta_5 atoms as a function of the average metal-oxygen bond lengths.

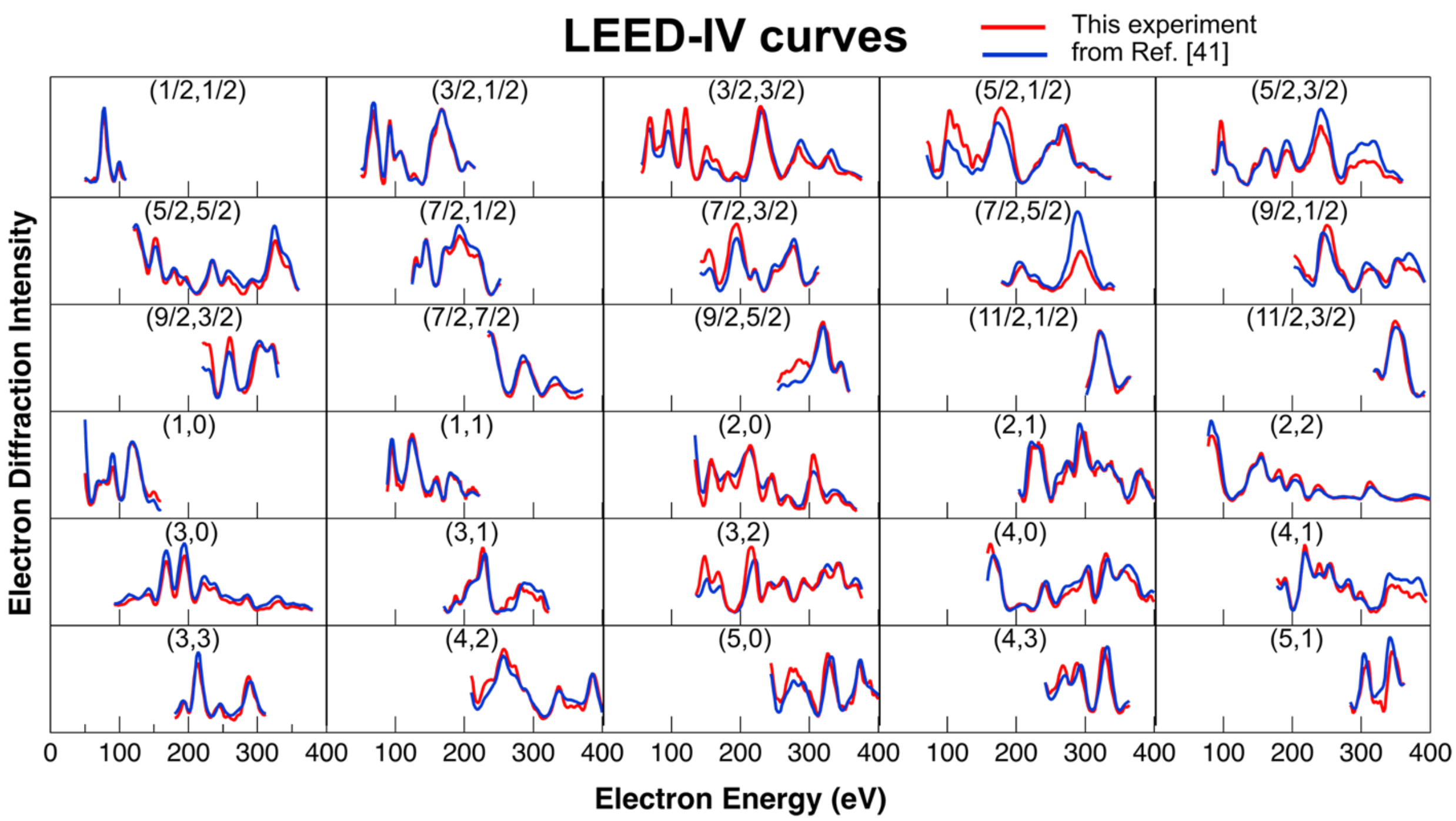

Figure 6S. Comparison of computed LEED-IV curves from LEED pattern acquired in this experiment (red curves) and those computed by Parkinson's group (blue curves).

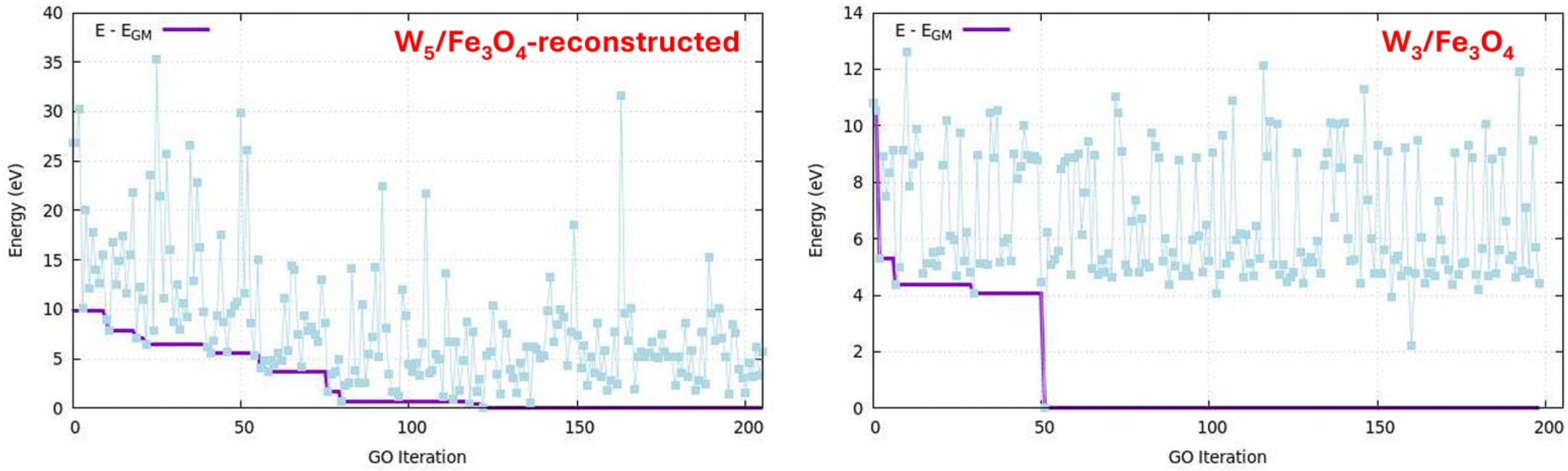

Figure 7S. Energy evolution as a function of the Basin-hopping iteration for representative Fe_3O_4/W systems.
